# Physicians’ behavior regarding non-acceptance of oral restriction (*nil per os*) by dysphagic patient with risk of laryngotracheal aspiration

**DOI:** 10.31744/einstein_journal/2020AO4952

**Published:** 2019-12-20

**Authors:** Frederico de Lima Alvarenga, Leonardo Haddad, Daniel Marcus San da Silva, Eliézia Helena de Lima Alvarenga

**Affiliations:** 1 Faculdade de Medicina de Jundiaí, Jundiaí, SP, Brazil; 2 Escola Paulista de Medicina, Universidade Federal de São Paulo, São Paulo, SP, Brazil

**Keywords:** Deglutition disorders, Bioethics, Dysphagia, Aged, Personal autonomy

## Abstract

**Objective:**

To define physician´s behavior in the face of a mentally capable elderly dysphagic patients at risk of pulmonary aspiration, who do not accept oral restriction.

**Methods:**

Observational, cross-sectional study, presenting a clinical case of an independent elderly with clinical complaints of dysphagia and laryngotracheal aspiration by flexible endoscopic evaluation of swallowing who rejected the proposal to restrict oral diet. A questionnaire about the patient's decision-making process was used to assess whether the physician was sympathetic and justify their answer, and if they are aware of hierarchy of ethical principles (recognition of the person´s value, autonomy, beneficence, nonmaleficence and justice), in the decision-making process, and which was the main principle that guided their decision.

**Results:**

One hundred participants were classified by time since graduation as Group I (less than 10 years) and Group II (more than 10 years). Of them, 60% agreed with the patient's decision, with no difference between the groups. The main reason was autonomy of patients, in both groups. Among those who were not sympathetic, the main argument was beneficence and nonmaleficence, considering the risk between benefit and harm. As to awareness about the hierarchy of principles, we did not find differences between the groups. Autonomy was the principle that guided those who were sympathetic with the patient's decision, and justice among those who didnot agree.

**Conclusion:**

Physicians were sympathetic with the patient's decision regarding autonomy, despite the balance between risks of beneficence and nonmaleficence, including death. We propose to formalize a non-compliance term.

## INTRODUCTION

Dysphagia is any difficulty in the swallowing process, in the transport of food from the mouth to the stomach,^(^^1^^)^ whether due to anatomofunctional modification or neuromuscular impairment. This condition may result in the interruption of the pleasure of eating, because of its repercussions in the safety and comfort of feeding, in nutrition and hydration, and even in the control of oral secretions, leading to changes in habits, and often, in oral feeding restrictions and resulting decline in quality of life.^(^^2^^,^^3^^)^

Due to the high risk of aspiration caused by dysphagia, it is difficult to distinguish the fine line that separates dysphagia from aspirative syndromes, which are frequently silent and increase the risk of aspiration pneumonia, as well as to structural and functional impairment of the lungs, deteriorating the fragile health of the elderly, and increasing morbidity and mortality.^(^^1^^)^ Dysphagia has become an emergent concern among healthcare professionals, especially those who treat elderly patients, with an impact on quality of life, and tends to be a public health problem.^(^^4^^–^^7^^)^

The prevalence of dysphagia varies according to the group studied: 13% of individuals aged 65 years, 16% in the age group 70-79 years, and 33% among individuals over 80 years of age who live independently.^(^^4^^,^^8^^)^

The physician-patient relationship is ruled by ethical bases and principles,^(^^9^^,^^10^^)^ seeking to value human beings (integrity/dignity); to do good (maximize the benefit) − beneficence, and avoid evil (the physician's actions should always cause the least hindrance to the patient's health) – nonmaleficence; respecting the patient's choices (autonomy); and seeking to be just in every action (justice).

Considering the impossibility of oral ingestion due to the risks exposed above, a proposal of oral route restriction, with indication of the nasogastric tube (NGT) or percutaneous endoscopic gastrostomy (PEG) is made for the patient. This management may be promptly accepted or not. In face of the refusal by the patient, we should clarify the inherent risks of maintenance of the oral route, and with additional information, the patient and their family members can accept such a proposal. The professional should make every effort to explain its importance, based on the principle of beneficence and not of autonomy.

The current study is justified based on the following dilemma: a patient who is at risk of pulmonary aspiration when feeding, but insists on feeding him/herself orally, refusing the proposed procedure of an alternate route to guarantee water and calorie intake, considering that he/she is malnourished, and running the risk of aspiration.

## OBJECTIVE

To analyze medical behavior in case of refusal of proposal of oral intake restriction in the dysphagic patient with risk of pulmonary aspiration, in the light of bioethics.

## METHODS

This is an observational study carried out from September to November 2017. During the recruitment process, we presented the study design to attending physicians from different organizations in the city of São Paulo. Those interested in participating voluntarily were included and invited to sign the Informed Consent Form (ICF). The study was approved by the Ethics Committee of *Hospital São Paulo* and was registered at *Plataforma Brasil*, with certificate of presentation for ethical appreciation, CAAE: 78089717.8.00005505 and opinion no. 2.423.709.

We present a true case of a lucid mentally capable elderly 86-year-old patient, who lived independently, with an active social life, who had had difficulty swallowing every food consistency over the previous two years, with non-intentional weight loss of 12kg (almost 20% of body weight), choking on his own saliva, symptoms that worsened with the offer of food, throat clearing and cough. He denied pneumonia but had past history of ischemic stroke one year before, without sequelae. He did not accept changes in diet or speech therapy.

**Figure 1:**
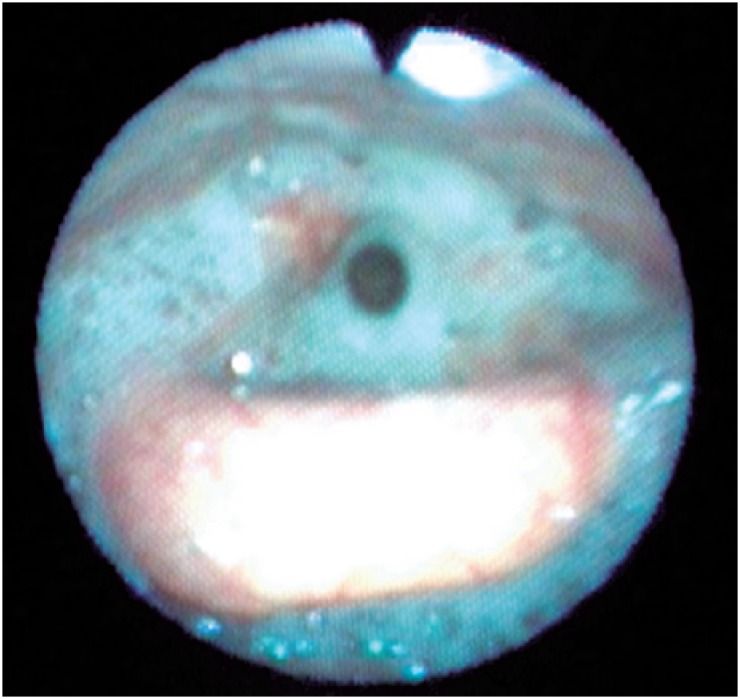
Flexible endoscopic evaluation of swallowing showed stasis of saliva in valleculae, pyriform sinuses, and retrocricoid region, with laryngotracheal penetration and aspiration

**Figure 2:**
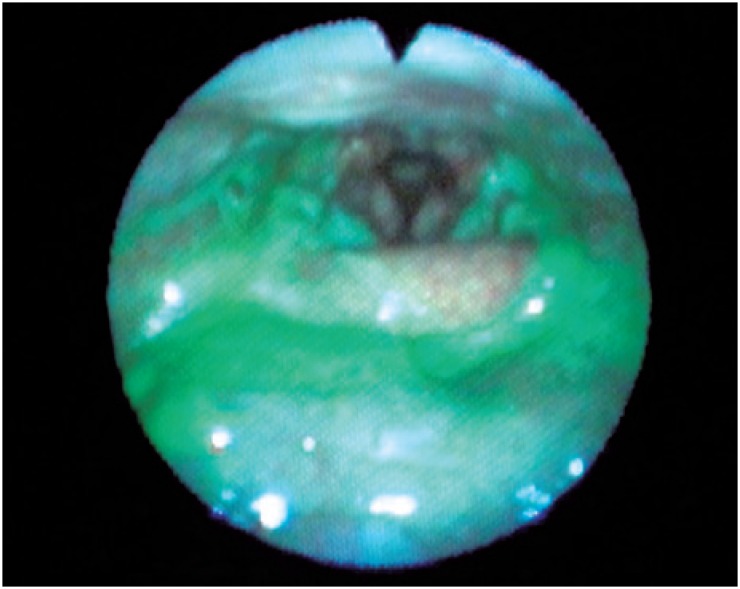
Food residue in valleculae, pyriform sinuses, and post-cricoid region, with laryngotracheal penetration and aspiration with the attempt to throat clearing the glottal, but without success

A flexible endoscopic evaluation of swallowing (FEES) was performed, which showed saliva stasis in valleculae, pyriform sinuses, and retrocricoid region, with laryngotracheal penetration and aspiration, and insufficient throat clearing and cough to clear glottis (Figure 1). The offer of all food consistencies, such as soft, liquid, and solid, showed residue of food after the third spontaneous swallow, penetration, and laringotracheal aspiration (Figure 2) with the effort to throat clearing the glottal chink, also without success. Additionally, we observed decreased pharyngeal constriction and the presence of laryngeal sensitivity. There was improvement with facilitating maneuvers to throat clearing, and swallow in sequence, chink tuck and multiple swallows, but insufficient for guaranteeing eating safety. Thus, the diagnosis was made of oropharyngeal dysphagia, with risk of laryngothracheal aspiration, malnutrition, and dehydration. An alternative feeding route was proposed by using a NGT, aiming at hydration and nutrition for weight recovery, in addition to speech therapy for reestablishing safe swallowing. The patient did not accept the management suggested, despite all efforts to clarify the risks and benefits of the procedure.

We presented a written self-explanatory questionnaire to 100 physicians who could treat the dysphagic patient (ear-nose-throat ENT specialists, head and neck surgeons, general practitioners, pulmonologists, gastroenterologists, geriatricians, neurologists, and intensive care physicians), as to the patient's decision in light of bioethics. In this questionnaire, we evaluated: (1) whether the physician agreed or not with the patient's decision, and then, justification was requested of the answer, which was classified within the principles and foundations that rule the decision-making process (acknowledgment of the person's value − integrity/dignity; autonomy; beneficence; nonmaleficence; and justice), and (2) whether the participant had knowledge of the ethical principles in face of a decision-making process, and which was the primary principle that directed their decision. The proposed questionnaire was designed by the investigators.

### Statistical analysis

Sample calculation was made using the G-Power 3.1 program, with a 90% confidence level, composed of 100 patients. The characteristics evaluated were described according to the groups of time since graduation, and the existence of an association with the likelihood ratio test (LRT) was verified when we used the primary argument and the main principle. The χ^2^ test or the exact test (Fisher's exact test) was used in the analysis of hierarchy of principles.

For each group, the associations between agreement and the primary argument used in the decision were assessed, and we also considered the response of awareness of hierarchy, with classification of the main principle (excluding the remaining principles) utilized individually and voluntarily chosen, which rules the decision-making process in the management proposed with the use of the LRT. Moreover, the primary principle in hierarchy (not excluding the others) was assessed by the chi-squared test or Fisher's exact test in the analysis of hierarchy of principles. The tests were performed with a 5% significance level.

## RESULTS

The 100 participants were divided into two groups: Group I (GI), with physicians with up to 10 years since graduation (58%), in which 34 were residents exclusively at public services (20 in ENT and 14 from other specialties), and other classified individuals, such as fellows, collaborators, and lecturers (18 ENT specialists, and 6 from other specialties); and Group II (GII), composed of professionals with more than 10 years since graduation (42%), in which 19 were ENT professionals, and 23 were from other specialties. The remaining 66 participants worked both in the private and public areas. As to sex, 52% were female.

Table 1 classifies the answers to the questionnaires of 100 volunteer physicians divided into GI (58) and GII (42), as to being sympathetic with the decision made by the patient, 43 physicians (74.1%) from GI and 26 (61.9%) from GII were sympathetic, and this was the primary argument used for their decision. Table 1 shows that there was no statistically significant association between the groups as to being sympathetic (p=0.192) and the arguments utilized.

**Table 1:** Description of sympathy, and the main argument (principle) used in the decision, as per groups and result of tests of association

Variable		Total	Time since graduation		OR	95%CI		p value
			Up to 10 years	More than 10 years				
		(n=100)	(n=58)	(n=42)		Inferior	Superior	
Sympathetic								0.192*
	No	31 (31)	15 (25.9)	16 (38.1)				
	Yes	69 (69)	43 (74.1)	26 (61.9)	0.57	0.24	1.34	
Argument†								
	Autonomy	35 (36.1)	23 (41.8)	12 (28.6)	0.56	0.24	1.31	0.178*
	Autonomy *versus* do good *versus* avoid evil	33 (34.0)	14 (25.5)	19 (45.2)	2.42	1.03	5.71	0.042*
	Do good and avoid evil	26 (26.8)	16 (29.1)	10 (23.8)	0.76	0.30	1.91	0.561*
	Autonomy and dignity	3 (3.1)	2 (3.6)	1 (2.4)	0.65	0.06	7.38	>0.999‡

Results expressed as n (%).

* χ^2^ test;

† 3 people from the group with up to 10 years since graduation did not respond;

‡ Fisher's exact test.

OR: odds ratio; 95%CI: 95% confidence interval.

Nevertheless, the main argument to be sympathetic with the patient's decision was isolatedly autonomy, followed by the balance among the three principles (autonomy, beneficence, and nonmaleficence). Among the non- sympathetic, the argument used was the balance between the two principles: do good and avoid doing evil (p<0.001).

Table 2 shows the main principle of decision voluntarily chosen - to be or not sympathetic - between the two groups.

**Table 2:** Description of awareness relative to the hierarchy of principles that rule the decision, and the main principle used in the decision, as per groups and the results of the tests of association

Variable		Total	Time since graduation		OR	95%CI		p value
			Up to 10 years	More than 10 years				
		(n=100)	(n=58)	(n=42)		Inferior	Superior	
Awareness of hierarchy								0.764*
	No	47 (47)	28 (48.3)	19 (45.2)				
	Yes	53 (53)	30 (51.7)	23 (54.8)	1.13	0.51	2.51	
Hierarchy†								
	Autonomy	43 (43.4)	26 (44.8)	17 (41.5)	0.87	0.39	1.96	0.739*
	Dignity	9 (9.1)	6 (10.3)	3 (7.3)	0.68	0.16	2.91	0.732‡
	Beneficence	32 (32.3)	23 (39.7)	9 (22)	0.43	0.17	1.06	0.064*
	Justice	15 (15.2)	3 (5.2)	12 (29.3)	7.59	1.98	29.05	0.001*

Results expressed as n (%).

* χ^2^ test;

† one person of the group with more than 10 years since graduation did not hierarchize;

‡ Fisher's exact test.

OR: odds ratio; 95%CI: 95% confidence interval.

There was no statistically significant association between the groups as to awareness of hierarchy of principles that rule the decision (p=0.764), but in GII, justice as the main principle used was statistically more frequent when compared to GI (p=0.001).

Table 3 describes the main principle chosen by each participant, in the hierarchy of the decision process, according to sympathy in each group.

**Table 3:** Description of classification of the main principle in hierarchy, according to sympathy in each group per time since graduation and the result of association tests

		Group I						Group II					
Variable		Not sympathetic	Sympathetic	OR	95%CI		p value	Not sympathetic	Sympathetic	OR	95%CI		p value
		(n=15)	(n=43)		Inferior	Superior		(n=16)	(n=26)		Inferior	Superior	
Main principle in decision													
	Autonomy	6 (40.0)	20 (46.5)	1.30	0.40	4.31	0.662*	2 (12.5)	15 (60.0)	10.50	1.95	56.56	0.003*
	Dignity	1 (6.7)	5 (11.6)	1.84	0.20	17.18	>0.999†	1 (6.2)	2 (8.0)	1.30	0.11	15.69	>0.999†
	Beneficence	6 (40.0)	17 (39.5)	0.98	0.30	3.26	0.975*	4 (25.0)	5 (20.0)	0.75	0.17	3.35	0.717†
	Justice	2 (13.3)	1 (2.3)	0.16	0.01	1.85	0.161†	9 (56.2)	3 (12.0)	0.11	0.02	0.50	0.004†

Results expressed as n (%).

* χ^2^ test;

† Fisher's exact test.

OR: odds ratio; 95%CI: 95% confidence interval.

In GII, autonomy as the main principle in the decision was statistically greater in sympathetic individuals (p=0.003), while justice as the main principle in the decision was statistically more frequent in the not sympathetic (p=0.004).

There was no statistically significant association between awareness of hierarchy and the main principle chosen in the decision between the two groups (p>0.05).

We separately analyzed the group of ENTs, composed of 57 participants and divided into two groups: Group ENT I, for those graduated up to 10 years before (63%), and Group ENT II, for those who graduated over 10 years before (37%). The ENTs were residents, fellows, collaborators, and lecturers. The main argument used (autonomy) showed a statistically significant association with sympathy in both groups (p<0.001 and p=0.032, respectively, Group ENT I and Group ENT II). The main argument to be sympathetic with the patient's decision was isolatedly autonomy, followed by a balance among the three principles: autonomy, beneficence, and nonmaleficence. Among the not sympathetic, the argument used was the balance between the two principles: do good and avoid doing evil (p<0.001).

We evaluated the main principle of decision voluntarily chosen in the decision to be or not sympathetic between the two groups of ENTs, and whether they were aware of the hierarchy of principles. There was no statistically significant association between the groups as to awareness of hierarchy of principles that rule the decision (p=0.707), nor as to the main principle used in the individual's decision to be or not be sympathetic.

Among the ENT specialists, there was no statistically significant association between them as to awareness of hierarchy of principles that rule the decision (p=0.707) and the time since graduation, but in the ENT II Group, justice as the primary principle used was statistically more frequent compared to the ENT I Group (p=0.022). Applying Fisher's exact test to the hierarchization of principles, as primary principle in the decision, autonomy proved to be statistically higher in sympathetic individuals (p=0.003), and justice among the not sympathetic (p=0.004), ENT II Group.

Table 4 classifies the hierarchy of the principles that rule the medical decision as to whether or not be sympathetic with the patient's decision (in first place, not excluding the remaining principles) divided into two groups classified by time since graduation. Among the ENTs who graduated over 10 years before, autonomy as the primary principle was statistically higher than in those who considered themselves sympathetic (p=0.024).

**Table 4:** Description of the classification of the primary principle in the hierarchy, according to the sympathy, among ENT specialists in each group, classified by the time since graduation and the result of the tests of association

		Group ENT I						Group ENT II					
Variable		Not sympathetic	Sympathetic	OR	95%CI		p value	Not sympathetic	Sympathetic	OR	95%CI		p value
		(n=10)	(n=26)		Inferior	Superior		(n=10)	(n=11)		Inferior	Superior	
Main principal in the decision													
	Autonomy	3 (30)	14 (53.8)	2.72†	0.57	12.91	0.274*	1 (10)	7 (63.6)	15.75	1.42	174.25	0.024*
	Dignity	0 (0)	3 (11.5)				0.545*	2 (20)	1 (9.1)	0.40	0.03	5.25	0.586*
	Beneficence	6 (60)	9 (34.6)	0.35†	0.08	1.58	0.260*	3 (30)	2 (18.2)	0.52	0.07	4.00	0.635*
	Justice	1 (10)	0 (0)				0.278*	4 (40)	1 (9.1)	0.15	0.01	1.68	0.149*

Results expressed as n (%).

* Fisher's exact test;

† there are no cases to estimate.

OR: odds ratio; 95%CI: 95% confidence interval.

## DISCUSSION

Among the greatest challenges in the management of dysphagia are the legal, ethical, and moral needs of the requirement to *nil per os* and propose feeding by an alternative route, via NGT or PEG. This is to guarantee fluid and nutrition support and minimize the risk of pulmonary aspiration until reestablishing safe swallowing, which must have the consent from the patient. When this management is not accepted by the patient, a dilemma appears, and the main objective of the study was that of defining the physician's behavior, in light of bioethics, in face of the refusal of a proposal of *nil per os* in the dysphagic patient with risk of pulmonary aspiration.

Nutrition is a basic human right that simply cannot be denied,^(^^11^^)^ and there is a proposal that malnutrition be included among the giants of the geriatric syndrome,^(^^12^^)^ since the relation between oropharyngeal dysphagia and increased morbidity and mortality in hospitalized patients is well established.^(^^13^^,^^14^^)^ The European Society for Swallowing Disorders (ESSD) defines guidelines for the evaluation and safety of swallowing, and determined the nutritional support be assured, until recovery of the nutritional status and rehabilitation of swallowing.^(^^15^^)^ If malnutrition is present, an individualized program should be developed, considering different aspects of the aged, such as beliefs, attitudes, preferences, expectations, and aspirations.^(^^16^^)^

To obligate the patient, however, to receive feeding through an alternative route against his/her desire is an object of discussion. Some consider it the means to preserve life, morally required to guarantee human dignity and integrity;^(^^11^^)^ and a duty for the protection of frail elderly individuals with dementia. To others, it is a lack of respect of the right to chose (autonomy) when refusing the therapeutic response at issue,^(^^12^^)^ even among the aged and frail, but with cognition intact.^(^^11^^)^ Our study showed that the analysis among physicians sympathetic with the dysphagic patient's decision in refusing feeding by an alternative route, despite the risk of pulmonary aspiration, was respect for the autonomy of decision, followed by the balance among the three principles: autonomy, beneficence, and nonmaleficence. The same was found among the ENT specialists.

Information should be clear and sufficient in order to guarantee that the patients understand the medical decision, considering the duty to care and the interests of the patient. We suggest a broad discussion of the risks and benefits of the proposal, the risk of not accepting the medical management, and the search for consent, in face of an individual who is mentally capable. There should also be the right to change opinions at any time, both in favor of and contrary to the management proposed. The autonomy of the capable patient should be respected, even if the refusal might result in death. This was well established by Law 10.241 and was discussed in patient rights and legislations.^(^^10^^)^ The best way to improve patients’ autonomy is by providing information and sharing decision making.^(^^11^^,^^12^^,^^17^^)^

Among the non-sympathetic, the primary argument used was the balance between the two principles: do good and avoid evil.

Bioethical principles are applied to swallowing disorders and decision-making in clinical practice. Beneficence means to “do good,” and nonmaleficence means “avoid evil,” guaranteeing respect for the patient's autonomy and justice.^(^^12^^,^^17^^)^ We chose to include the foundation of integrity and dignity in our questionnaire. Whenever the professional proposes any therapeutic intervention, first of all, we should recognize the dignity of the patient and consider them in their totality (all dimensions of human beings should be considered: physical, psychological, social, and spiritual), seeking to offer them the best treatment, both as to technique and as to acknowledgement of the physical, psychological, or social needs.

A professional should, above all, desire the best for the patient (beneficence), in order to reestablish health, prevent worsening, or promote health. In the case at issue, this would result in proposing effective treatment for dysphagia, aiming at nutritional recovery, guaranteeing nutritional and water intake, and restricting the oral route until swallowing is rehabilitated by means of speech therapy.^(^^15^^)^

We perceive that there is difficulty in considering these two principles separately (beneficence and nonmaleficence), because we always evaluate the bionomial as a balance between risk and benefit (do good and avoid evil) of any proposal in medical care. On the other hand, when the results are uncertain and it is improbable that damage be done (avoid evil - nonmaleficence), abstention from treatment is justifiable for reasons that are both moral and ethical. In the aged, the application of the principle of beneficence should override all other considerations, as per Eibling et al.^(^^6^^)^

Intuitively, physicians know how to argue about their position in being sympathetic or not with the patient's decision to refuse the medical management. Generally, their answers are based on the principles, but not all patients are aware of hierarchy of principles, and even those who do know of its existence, do not necessarily base themselves on the primary hierarchical principle as being the main principle, nor do they show a relation between awareness of hierarchy with time since graduation. Considering the hierarchization of the principles, as the first principle in the decision, autonomy was shown to be more frequent among sympathetic physicians (according to the hierarchization of principles: the first/primary and main principle), and justice among those who are not sympathetic (although hierarchically it is the last principle) in GII. The same was found among the ENT specialists.

Patients should be treated justly, with no discrimination based on social status, race, ethnicity, or religious beliefs. In practice, healthcare professionals need to develop sensitivity towards diversity of opinion. The principle of justice overlapped among the physicians with more time since graduation; justified by the idea that a decision would not be ethical if one of the players involved (professional or patient) were to be harmed. This idea is also reinforced among those who were not sympathetic, within this context of giving the person what he/she needs.

Oral ingestion is a complex process vital for life. It is not only essential for existence, but also plays an important role in happiness and socialization, and restriction of the oral route reflects on pleasure/happiness and consequently, on quality of life, leading us to ethical considerations. For some patients, there is loss of quality of life, damaging the main principle, which is integrity/dignity.^(^^11^^)^

Conscious of the unique capacity of human being to reflect on their own existence and on their environment; perceive injustice; avoid danger; take responsibility; seek cooperation, and demonstrate the moral meaning that provides the expression of ethical principles, so that we should always seek their consent.^(^^11^^)^

Patients who presented with unsafe swallowing, but have mental capacity, should be encouraged to follow the recommendations of the physician and initiate speech therapy in order to maintain the oral route, rehabilitating it as quickly as possible, not those who remain in *nil per os,* that is, nothing by oral route. In this way, the management of the oral offer by caregivers and/or family members, under some conditions, a diet may be offered with modified texture, or have restrictions of one or more food consistencies, according to the medical and speech therapy evaluations.^(^^15^^)^

The risk of autonomy should be clarified. The patient is not always in a condition to evaluate which is the best treatment for them (after all, they are laypeople and do not have the technical knowledge necessary for this). The professional should make every possible effort to explain to the patient the importance of the management plan. After all, it is the principle of always seeking consent, making clear the possibility of patients to change their mind. The patient described herein, for example, returned to the physician who had cared for him convinced of the proposed management, and he was given feeding by NGT and speech therapy. He recovered his nutritional status and safe oral feeding.

One of the limitations of this study is our sample, with a 90% confidence power. Since it is an observational study, it allows the establishment of associations, but does not define the relation of causality and risk. We highlight its clinical importance in the need to initiate a wider discussion among all medical specialties as to the decision of the patient who does not accept our management, discuss the bioethical principles, learn to conduct the cases that challenge us, and document the patient's refusal. We should not back down on encouraging the patient with information about their true health status and their expectations in face of non-acceptance of the treatment proposed, and the consequences of refusal, since many times, the autonomy of the patient is distorted by misinformation, and doubts about the therapeutic proposal, which once clarified, could change their position. We need to attempt to convince the patients of the best, and if not, give them support to guide them in face of the refusal.

## CONCLUSION

In face of the mentally capable dysphagic patients with risk of laryngotracheal aspiration who says that they do not accept restriction of the oral route NPO, the physician faces a conflict of ethical and moral values. Our study demonstrated that the majority of participants were sympathetic with the patient's decision and argued that autonomy is isolatedly sovereign and/or despite the risks, balance between the principles of beneficence and nonmaleficence. Additionally, the decision should be respected in individuals who are mentally capable, as well as the right to change opinion. Among the non-sympathetic participants, the balance between the principles of beneficence and nonmaleficence guided their decision, justifying the risk of clinical worsening with increased morbidity and mortality. Regardless of awareness of hierarchy of the principles that orient the decision process in our sample, the primary principle considered in this process was that of autonomy among the participants sympathetic with the decision contrary to the proposal offered. The principle of justice predominated among the non-sympathetic participants with more time since graduation. Ethical concerns arise of non-acceptance of the action proposed, and it is necessary to formalize the refusal in the medical record, by means of an informed refusal document, with legal protective purposes.
