# Supplementary material for: Physicians’ behavior regarding non-acceptance of oral restriction (nil per os) by dysphagic patient with risk of laryngotracheal aspiration
Source: Einstein (Sao Paulo). 2019 Dec 20;18:eAO4952. doi: 10.31744/einstein_journal/2020AO4952 (PMC6924824; doi:10.31744/einstein_journal/2020AO4952)
Supplement: Translation

# Translation: Comportamento do médico diante da não aceitação de restrição da via oral pelo paciente disfágico com risco de aspiração laringotraqueal

**Authors:** Frederico de Lima Alvarenga, Leonardo Haddad, Daniel Marcus San da Silva, Eliézia Helena de Lima Alvarenga

**Affiliations:** 1 Faculdade de Medicina de Jundiaí, Jundiaí, SP, Brasil; 2 Escola Paulista de Medicina, Universidade Federal de São Paulo, São Paulo, SP, Brasil

**Keywords:** Transtornos de deglutição, Bioética, Disfagia, Idoso, Autonomia pessoal

## Abstract

**Objetivo:**

Definir o comportamento médico diante de paciente idoso disfágico com risco de aspiração laringotraqueal e mentalmente capaz que não aceita a restrição da via oral.

**Métodos:**

Estudo observacional, transversal. Apresentamos um caso clínico de idoso, que vive independente, com queixas clínicas e videoendoscopia da deglutição comprovando disfagia e aspiração, que recusou a proposta de restrição da via oral. Um questionário foi aplicado sobre o processo de decisão do paciente, procurando avaliar se o médico torna-se solidário, e que justifique sua resposta, e se tem ciência da hierarquia dos princípios éticos (reconhecimento do valor da pessoa, autonomia, beneficência, não maleficência e justiça), no processo de decisão e qual o principal princípio que norteia sua decisão.

**Resultados:**

Cem participantes foram classificados por tempo de formados em Grupo I (até 10 anos) e Grupo II (mais de 10 anos). Deles 60% tornaram-se solidários à decisão do paciente, sem diferença entre os grupos. O principal argumento foi a autonomia do paciente nos dois grupos. Entre os não solidários, foi o binômio beneficência e não maleficência, e o balanço do risco/benefício e malefício. Considerando a ciência sobre a hierarquia dos princípios que regem a decisão, não encontramos diferença entre eles. A autonomia foi o principal princípio na decisão entre os solidários e a justiça entre os não solidários.

**Conclusão:**

O médico foi solidário à decisão do paciente em respeito à sua autonomia, apesar dos riscos ponderados da beneficência e da maleficência, inclusive de morte. Propomos o termo de recusa de conduta formalizada.

## INTRODUÇÃO

Disfagia é qualquer dificuldade no processo de deglutição, no transporte do bolo alimentar da boca ao estômago,^(^^1^^)^ seja por alteração anatomofuncional, ou por comprometimento neuromuscular. Esta condição pode resultar na interrupção do prazer de se alimentar, por suas repercussões na segurança e conforto da alimentação, na nutrição e hidratação, e até mesmo no controle das secreções orais, implicando em mudança de hábitos e, algumas vezes, em restrição da alimentação por via oral e no consequente declínio da qualidade de vida.^(^^2^^,^^3^^)^

Pelo alto risco de aspiração causada pela disfagia, é difícil distinguir a linha tênue que separa disfagia das síndromes aspirativas, muitas vezes silenciosas e que predispõem não apenas à pneumonia, mas também ao comprometimento estrutural e funcional dos pulmões, deteriorando o estado frágil de saúde dos idosos; aumentando a morbimortalidade.^(^^1^^)^ A disfagia tem se tornado preocupação emergente entre os profissionais de saúde, especialmente aqueles que tratam pacientes idosos, com impacto na qualidade de vida e tendendo a ser um problema de saúde pública.^(^^4^^–^^7^^)^

A prevalência da disfagia varia de acordo com o grupo estudado: 13% dos indivíduos com 65 anos de idade, 16% na faixa etária de 70-79 anos e 33% entre os indivíduos acima de 80 anos que vivem de forma independente.^(^^4^^,^^8^^)^

A relação médico-paciente é regida por fundamentos e princípios éticos,^(^^9^^,^^10^^)^ visando à valorização do ser humano (integridade/dignidade); à busca por fazer o bem (maximizar o benefício) −beneficência, e evitar o mal (a ação do médico sempre deve causar o menor prejuízo à saúde do paciente) −não maleficência; respeitando as escolhas do paciente (autonomia); e procurando ser justos em todo ato (justiça).

Diante da impossibilidade da ingesta oral pelos riscos acima expostos, uma proposta de restrição da via oral, com indicação de sonda nasoenteral (SNE) ou gastrostomia (GTM) é feita ao paciente. Esta conduta pode ser prontamente aceita ou não. Diante da recusa pelo paciente, devemos esclarecer os riscos inerentes da manutenção da via oral e, com adicionais informações, o paciente e seus familiares podem aceitar tal proposta. O profissional deve se esforçar ao máximo para explicar a importância, baseado no princípio da beneficência e não da autonomia.

O atual estudo se justifica baseado no seguinte dilema: paciente que está em risco de broncoaspirar quando se alimenta, mas insiste em se alimentar por via oral, recusando a conduta proposta de via alternativa para garantir aporte hídrico e calórico, visto que está desnutrido e assumindo o risco de aspirar.

## OBJETIVO

Analisar o comportamento médico diante da recusa da proposta de restrição de via oral no paciente disfágico com risco de broncoaspiração à luz da bioética.

## MÉTODOS

Trata-se de estudo observacional, realizado de setembro a novembro de 2017. Durante o processo de recrutamento, apresentamos o desenho do nosso estudo aos médicos assistencialistas de diferentes instituições na cidade de São Paulo. Aqueles interessados em participar voluntariamente foram incluídos e convidados a assinar o Termo de Consentimento Livre e Esclarecido (TCLE). O estudo foi aprovado pelo Comitê de Ética do Hospital São Paulo e está registrado na Plataforma Brasil, sob certificado de apresentação para apreciação ética, CAAE: 78089717.8.00005505 e parecer 2.423.709.

Apresentamos um caso real, de idoso de 86 anos, lúcido, que vivia de forma independente, com vida social ativa, mentalmente capaz, com dificuldade para deglutir todas as consistências alimentares nos últimos 2 anos, com perda ponderal não intencional de 12kg (quase 20% do peso corporal), engasgos com a própria saliva e piores com a oferta alimentar, pigarro e tosse. Negava pneumonia, referia antecedente de acidente vascular encefálico isquêmico há 1 ano, sem sequelas. Não aceitava mudança de dieta, nem fonoterapia.

Realizada videoendoscopia da deglutição, que mostrou estase de saliva em valéculas, seios piriformes e região retrocricoídea, com penetração e aspiração laringotraqueal, com tentativa de limpeza da fenda glótica, mas sem sucesso (Figura 1). A oferta de todas as consistências alimentares, como pastoso, líquido e sólido, mostrou resíduo de alimento após a terceira deglutição espontânea, penetração e aspiração laringotraqueal (Figura 2), com tentativa de limpeza da fenda glótica também sem sucesso. Além disso, observaram-se constricção faríngea diminuída e sensibilidade laríngea presente. Houve melhora com as manobras facilitadoras de limpeza da fenda glótica e as supraglótica, mas não suficientes para garantir segurança da alimentação. Então, foi feito o diagnóstico de disfagia orofaríngea, com risco de broncoaspiração, desnutrição e desidratação. Foi proposta a via alternativa de alimentação por SNE, visando à hidratação e à nutrição para recuperação ponderal, além de fonoterapia para restabelecimento da segurança da deglutição. Paciente não aceitou a conduta sugerida, apesar de todos os esforços para esclarecer os riscos e os benefícios da conduta.

**Figura 1:** Videoendoscopia da deglutição: estase de saliva em valéculas, seios piriformes, e região retrocricoídea, com penetração e aspiração laringotraqueal

**Figura 2:** Resíduo de alimento em valéculas, seios piriformes e região retrocricoídea, com penetração e aspiração laringotraqueal, com tentativa de limpeza da fenda glótica, mas sem sucesso

Apresentamos um questionário autoexplicativo, por escrito, a cem médicos que podiam assistir o paciente disfágico (otorrinolaringologistas, cirurgiões de cabeça e pescoço, clínicos gerais, pneumologistas, gastrenterologistas, geriatras, neurologistas e intensivistas), sobre o processo de decisão do paciente à luz da bioética. Nesse questionário, foram avaliados: (1) se o médico era solidário ou não à decisão do paciente e, em seguida, foi solicitado para justificar sua resposta, que foi enquadrada dentro dos princípios e fundamentos que regem o processo de decisão (reconhecimento do valor da pessoa − integridade/dignidade; autonomia; beneficência; não maleficência; e justiça), e (2) se o participante tinha ou não ciência da hierarquia dos princípios éticos diante de um processo de decisão, e qual o principal princípio que norte avasua decisão. O questionário proposto foi desenhado pelos próprios pesquisadores

### Análise estatística

O cálculo amostral foi calculado usando o programa G-Power 3.1, com confiança de 90%, composto por cem pacientes.

Foram descritas as características avaliadas segundo grupos de tempo de formação e foi verificada a existência de associação com o teste da razão de verossimilhanças (TRV) quando utilizamos o principal argumento e o principal princípio, e utilizamos o teste χ^2^ ou teste exato (teste exato de Fisher) na análise da hierarquia dos princípios.

Para cada grupo, foram avaliadas as associações da solidariedade e o principal argumento utilizado na decisão, e consideramos também a resposta de ciência da hierarquia, com a ordenação do principal princípio (excludente dos demais princípios) utilizado individualmente e voluntariamente escolhido, que rege o processo de decisão na conduta proposta com uso de TRV, e o primeiro princípio na hierarquia (não excludente dos demais) com uso do teste χ^2^ ou teste exato de Fisher na análise da hierarquia dos princípios. Os testes foram realizados com nível de significância de 5%.

## RESULTADOS

Os 100 participantes foram divididos em 2 grupos: Grupo I (GI), com médicos com até 10 anos de formados (58%), sendo 34 residentes de serviço público exclusivo (20 da otorrinolaringologia e 14 de outras especialidades), e demais classificados como *fellows*, colaboradores, e docentes (18 otorrinolaringologistas, e 6 de outras especialidades); e Grupo II (GII), composto por profissionais com mais de 10 anos de formados (42%),sendo 19 otorrinolaringologistas e 23 de outras especialidades. Os demais 66 participantes prestavam assistência tanto na área privada como pública. Quanto ao sexo,52% eram mulheres.

A tabela 1 classifica as respostas do questionário de cem médicos voluntários divididos em GI (58) e em GII (42), em relação a solidariedade à decisão tomada pelo paciente: 43 médicos (74,1%) do GI e 26 (61,9%) do GII tornaram-se solidários, e o principal argumento utilizado para sua decisão.

**Tabela 1:** Descrição da solidariedade, e o principal argumento (princípio) utilizado em sua decisão, segundo grupos e resultado dos testes de associação

Variável		Total	Tempo de formado		OR	IC95%		Valor de p
			Até 10 anos	Mais de 10 anos				
		(n=100)	(n=58)	(n=42)		Inferior	Superior	
Solidário								0,192*
	Não	31 (31)	15 (25,9)	16 (38,1)				
	Sim	69 (69)	43 (74,1)	26 (61,9)	0,57	0,24	1,34	
Argumento†								
	Autonomia	35 (36,1)	23 (41,8)	12 (28,6)	0,56	0,24	1,31	0,178*
	Autonomiax fazer o bem x evitar o mal	33 (34,0)	14 (25,5)	19 (45,2)	2,42	1,03	5,71	0,042*
	Fazer o bem e evitar o mal	26 (26,8)	16 (29,1)	10 (23,8)	0,76	0,30	1,91	0,561*
	Autonomia e dignidade	3 (3,1)	2 (3,6)	1 (2,4)	0,65	0,06	7,38	>0,999‡

Resultados expressos como n (%).

* teste do χ^2^;

† 3 pessoas do grupo com até 10 anos de formado não responderam;

‡ teste exato de Fisher.

OR: *odds ratio*; IC95%: intervalo de confiança de 95%.

A tabela 1 mostrou que não houve associação estatisticamente significativa entre os grupos em relação à solidariedade (p=0,192) e aos argumentos utilizados.

Porém, o principal argumento para se tornar solidário à decisão do paciente foi a autonomia isoladamente, seguido do balanço entre os três princípios (autonomia, beneficência e não maleficência). Entre os não solidários, o argumento utilizado foi o balanço entre os dois princípios: fazer o bem e evitar fazer o mal (p<0,001).

A tabela 2 mostra o principal princípio de decisão voluntariamente escolhido na decisão de ser ou não solidário entre os dois grupos.

**Tabela 2:** Descrição da ciência em relação à hierarquia dos princípios que regem a decisão, e o principal princípio utilizado em sua decisão, segundo grupos e resultado dos testes de associação

Variável		Total	Tempo de formado		OR	IC95%		Valor de p
			Até 10 anos	Mais de 10 anos				
		(n=100)	(n=58)	(n=42)		Inferior	Superior	
Ciência da hierarquia								0,764*
	Não	47 (47)	28 (48,3)	19 (45,2)				
	Sim	53 (53)	30 (51,7)	23 (54,8)	1,13	0,51	2,51	
Hierarquia†								
	Autonomia	43 (43,4)	26 (44,8)	17 (41,5)	0,87	0,39	1,96	0,739*
	Dignidade	9 (9,1)	6 (10,3)	3 (7,3)	0,68	0,16	2,91	0,732‡
	Beneficência	32 (32,3)	23 (39,7)	9 (22)	0,43	0,17	1,06	0,064*
	Justiça	15 (15,2)	3 (5,2)	12 (29,3)	7,59	1,98	29,05	0,001*

Resultados expressos como n (%).

* teste do χ^2^;

† uma pessoa do grupo com mais de 10 anos de formado não hierarquizou;

‡ teste exato de Fisher.

OR: *odds ratio*; IC95%: intervalo de confiança de 95%.

Não houve associação estatisticamente significativa entre os grupos em relação à ciência da hierarquia dos princípios que regem a decisão (p=0,764), mas, no GII, a justiça como principal princípio utilizado foi estatisticamente mais frequente comparada ao GI (p=0,001).

A tabela 3 descreve o principal princípio escolhido por cada participante, na hierarquia do processo de decisão, segundo solidariedade em cada grupo.

**Tabela 3:** Descrição da ordenação do principal princípio na hierarquia segundo solidariedade em cada grupo por tempo de formação e resultado dos testes de associação

		Grupo I						Grupo II					
Variável		Não solidário	Solidário	OR	IC95%		Valor de p	Não solidário	Solidário	OR	IC95%		Valor de p
		(n=15)	(n=43)		Inferior	Superior		(n=16)	(n=26)		Inferior	Superior	
Principal princípio na decisão													
	Autonomia	6 (40,0)	20 (46,5)	1,30	0,40	4,31	0,662*	2 (12,5)	15 (60,0)	10,50	1,95	56,56	0,003*
	Dignidade	1 (6,7)	5 (11,6)	1,84	0,20	17,18	>0,999†	1 (6,2)	2 (8,0)	1,30	0,11	15,69	>0,999†
	Beneficência	6 (40,0)	17 (39,5)	0,98	0,30	3,26	0,975*	4 (25,0)	5 (20,0)	0,75	0,17	3,35	0,717†
	Justiça	2 (13,3)	1 (2,3)	0,16	0,01	1,85	0,161†	9 (56,2)	3 (12,0)	0,11	0,02	0,50	0,004†

Resultados expressos como n (%).

* teste do χ^2^;

† teste exato de Fisher.

OR: *odds ratio*; IC95%: intervalo de confiança de 95%.

No GII, a autonomia como principal princípio na decisão foi estatisticamente maior nas pessoas solidárias (p=0,003), enquanto a justiça como principal princípio na decisão foi estatisticamente mais frequente nos não solidários (p=0,004).

Não houve associação estatisticamente significativa da ciência da hierarquia com o principal princípio escolhido na decisão entre os dois grupos (p>0,05).

Analisamos em separado o grupo dos otorrinolaringlogistas, composto por 57 participantes, divididos em dois grupos: Grupo Otorrino I, para aqueles até 10 anos de formados (63%), e Grupo Otorrino II, para aqueles com mais de 10 anos de formados (37%). Os otorrinolaringologistas eram residentes, *fellows*, colaboradores e docentes. O principal argumento utilizado (autonomia) apresentou associação estatisticamente significativa com a solidariedade em ambos os grupos (p<0,001 e p=0,032, respectivamente, Grupo Otorrino I e Grupo Otorrino II). O principal argumento para se tornar solidário à decisão do paciente foi a autonomia isoladamente, seguida do balanço entre os três princípios: autonomia, beneficência e não maleficência. Entre os não solidários, o argumento utilizado foi o balanço entre os dois princípios: fazer o bem e evitar fazer o mal (p<0,001).

Avaliamos o principal princípio de decisão voluntariamente escolhido na decisão ser ou não solidário entre os dois grupos de otorrinolaringologistas, e se tinham ciência da hierarquia dos princípios. Não houve associação estatisticamente significativa entre os grupos em relação à ciência sobre a hierarquia dos princípios que regem a decisão (p=0,707), nem quanto ao principal princípio utilizado em sua tomada de decisão de ser ou não solidário.

Entre os otorrinolaringologistas, não houve associação estatisticamente significativa entre eles, em relação à ciência da hierarquia dos princípios que regem a decisão (p=0,707) e ao tempo de formado, mas, no G Otorrino II, a justiça como primeiro princípio utilizado foi estatisticamente mais frequente comparada ao G Otorrino I (p=0,022). Aplicando o teste exato de Fisher na hierarquização dos princípios, como primeiro princípio na decisão, a autonomia mostrou-se estatisticamente maior nas pessoas solidárias (p=0,003), e a justiça entre os não solidários (p=0,004), G Otorrino II.

A tabela 4 classifica a hierarquia dos princípios que regem a decisão médica em ser ou não solidário à decisão do paciente (em primeiro lugar, não excluindo os demais princípios) divididos em dois grupos classificados por tempo de formado. Dentre os otorrinolaringologistas com mais de 10 anos de formados, a autonomia como primeiro princípio foi estatisticamente maior nos que se diziam solidários (p=0,024).

**Tabela 4:** Descrição da ordenação do primeiro princípio na hierarquia segundo solidariedade entre os otorrinolaringologistas em cada grupo classificado pelo tempo de formação e resultado dos testes de associação

		Grupo Otorrino I						Grupo Otorrino II					
Variável		Não solidário	Solidário	OR	IC95%		Valor de p	Não solidário	Solidário	OR	IC95%		Valor de p
		(n=10)	(n=26)		Inferior	Superior		(n=10)	(n=11)		Inferior	Superior	
Principal princípio na decisão													
	Autonomia	3 (30)	14 (53,8)	2,72†	0,57	12,91	0,274*	1 (10)	7 (63,6)	15,75	1,42	174,25	0,024*
	Dignidade	0 (0)	3 (11,5)				0,545*	2 (20)	1 (9,1)	0,40	0,03	5,25	0,586*
	Beneficência	6 (60)	9 (34,6)	0,35†	0,08	1,58	0,260*	3 (30)	2 (18,2)	0,52	0,07	4,00	0,635*
	Justiça	1 (10)	0 (0)				0,278*	4 (40)	1 (9,1)	0,15	0,01	1,68	0,149*

Resultados expressos como n (%).

* teste exato de Fisher;

† não há casos para estimar.

OR: *odds ratio*; IC95%: intervalo de confiança de 95%.

## DISCUSSÃO

Entre os desafios no manejo da disfagia, está a discussão legal, ética e moral da necessidade de restringir alimentação por via oral e propor alimentação por via alternativa, por SNE ou GTM, para garantir o suporte hídrico e nutricional, e minimizar o risco de broncoaspirar, até que se restabeleça a segurança da deglutição, que deve ter o consentimento do paciente. Quando esta conduta não é acatada pelo paciente, surge um dilema, e o principal objetivo do trabalho foi definir o comportamento médico diante da recusa da proposta de restrição de via oral no paciente disfágico com risco de broncoaspiração à luz da bioética.

A nutrição é um direito humano básico que simplesmente não pode ser negado^(^^11^^)^ e existe proposta de que a desnutrição seja incluída entre os gigantes da síndrome geriátrica,^(^^12^^)^ pois a relação entre disfagia orofaríngea e aumento da morbimortalidade em pacientes hospitalizados está bem estabelecida.^(^^13^^,^^14^^)^ A *European Society for Swallowing Disorders* (ESSD) definiu diretrizes para a avaliação e a segurança da deglutição, e determinou que o suporte nutricional seja assegurado, até que ocorram recuperação do estado nutricional e reabilitação da deglutição.^(^^15^^)^ Se a desnutrição estiver presente, programa individualizado deveria ser desenvolvido, considerando diferentes aspectos do idoso, como crenças, atitudes, preferências, expectativas e aspirações.^(^^16^^)^

Porém, obrigar o paciente a receber alimentação por via alternativa contrário à sua vontade é objeto de discussão. Alguns consideram o meio de preservar a vida, moralmente obrigatório para garantir a integridade e a dignidade humana;^(^^11^^)^ e dever de proteção aos idosos frágeis com demência. Para outros, é um desrespeito ao direito de escolha (autonomia) em recusar a proposta terapêutica em questão,^(^^12^^)^ mesmo entre os idosos fragilizados, mas com cognitivo preservado.^(^^11^^)^ Nosso trabalho mostrou que a análise entre os médicos solidários à decisão do paciente disfágico em recusar a alimentação por via alternativa, apesar dos riscos de broncoaspiração, foi o respeito pela autonomia de decisão, seguido pelo balanço entre os três princípios: autonomia, beneficência e não maleficência. O mesmo foi encontrado entre os otorrinolaringologistas.

As informações devem ser claras e suficientes, para garantir que os pacientes entendam a decisão médica, ponderando o dever de cuidar e o interesse do paciente. Propomos uma discussão ampla dos riscos e benefícios da proposta, do risco de não aceitar a conduta e a busca pelo consentimento, diante de um indivíduo mentalmente capaz. Deve haver ainda o direito de mudar de opinião a qualquer momento, tanto a favor como contrário à conduta proposta. A autonomia do paciente capaz deve ser respeitada, mesmo que a recusa possa resultar em morte, sendo bem estabelecida pela lei 10.241 e discutida em direitos dos pacientes e legislação.^(^^10^^)^ A melhor forma de aprimorar a autonomia dos pacientes é por meio da provisão de informações e tomada de decisão compartilhada.^(^^11^^,^^12^^,^^17^^)^

Entre os não solidários, o principal argumento utilizado foi o balanço entre os dois princípios: fazer o bem e evitar fazer o mal.

Os princípios bioéticos são aplicados aos distúrbios da deglutição e na tomada de decisões na prática clínica. Beneficência significa “fazer o bem”, e não maleficência significa “evitar o mal”, garantindo respeito pela autonomia do paciente e justiça.^(^^12^^,^^17^^)^ Optamos em incluir o fundamento da integridade e da dignidade no nosso questionário. Sempre que o profissional propuser qualquer intervenção terapêutica, primeiramente devemos reconhecer a dignidade do paciente e considerá-lo em sua totalidade (todas as dimensões do ser humano devem ser consideradas: física, psicológica, social e espiritual), visando oferecer-lhes o melhor tratamento, tanto no que diz respeito à técnica quanto no que se refere ao reconhecimento de suas necessidades físicas, psicológicas ou sociais.

Um profissional deve, acima de tudo, desejar o melhor para seu paciente (beneficência), para restabelecer sua saúde, prevenir um agravo ou promover sua saúde. No caso em questão, resultaria em propor tratamento efetivo para a disfagia, visando à recuperação nutricional, garantindo aporte nutricional e hídrico, e restringir a via oral, até que haja reabilitação da deglutição por meio da fonoterapia.^(^^15^^)^

Sentimos que há dificuldade em considerar em separados estes dois princípios (beneficência e não maleficência), porque sempre avaliamos o binômio – como em um balanço entre risco e benefício (fazer o bem e evitar o mal) de qualquer proposta em cuidados médicos. Por outro lado, quando os resultados são incertos e é provável que o dano seja causado (evitar o mal −não maleficência), a abstenção de tratamento é justificável tanto por razões morais quanto éticas. No idoso, a aplicação do princípio da beneficência deveria superar todas as outras considerações, de acordo com Eibling et al.^(^^6^^)^

Intuitivamente, o médico sabe argumentar sobre sua posição em se tornar ou não solidário à decisão do paciente de recusar a conduta médica. Geralmente suas respostas são pautadas nos princípios, mas nem todos têm ciência da existência de hierarquia dos princípios, e mesmo os que sabem de sua existência não necessariamente se baseiam no primeiro princípio hierárquico como o principal, nem mostram relação de ciência da hierarquia com o tempo de formado. Considerando a hierarquização dos princípios, como primeiro princípio na decisão, a autonomia mostrou-se mais frequente entre os médicos solidários (de acordo com a hierarquização dos princípios: o primeiro e principal), e a justiça entre os não solidários (embora seja o último princípio hierarquicamente) no GII. O mesmo foi encontrado entre os otorrinolaringologistas.

Os pacientes devem ser tratados de forma justa, sem discriminação com base no *status* social, raça, etnia ou crença religiosa. Na prática, os profissionais de saúde precisam desenvolver sensibilidade para a diversidade de opinião. O princípio da justiça sobrepôs entre os médicos com mais tempo de formado; justificado pela ideia que não seria ética uma decisão que levasse um dos personagens envolvidos (profissional ou paciente) a se prejudicar. Essa ideia também é reforçada entre aqueles que não se tornaram solidários, nesse contexto de dar a cada pessoa o que ela necessita.

A ingestão oral é um processo complexo essencial para a vida. Não só é essencial para a existência, mas também desempenha papel importante na felicidade e na socialização, e a restrição da via oral reflete no prazer/felicidade e, consequentemente, na qualidade de vida, provocando-nos reflexão ética. Para alguns pacientes, há perda da qualidade de vida, causando prejuízo ao principal fundamento, que é a integridade/dignidade.^(^^11^^)^

Consciente da capacidade única dos seres humanos de refletir sobre sua própria existência e sobre seu meio ambiente; perceber a injustiça; evitar o perigo; assumir responsabilidade; buscar cooperação e demonstrar o sentido moral que dá expressão a princípios éticos, de modo que devemos sempre procurar seu consentimento.^(^^11^^)^

Os pacientes que apresentam deglutição não segura, mas têm capacidade mental, devem ser encorajados a seguir o conselho médico e iniciar fonoterapia para manter a via oral, reabilitando mais rapidamente, do que aqueles que permanecem em *nil per os,* ou seja, nada por via oral. Assim, fica garantido o gerenciamento da oferta oral por cuidadores e/ou familiares e, em algumas condições, pode ser ofertada dieta com textura modificada, ou haver restrição de uma ou mais consistência de alimento, de acordo com a avaliação médica e fonoaudiológica.^(^^15^^)^

O risco da autonomia deve ser esclarecido. Nem sempre o paciente tem condições de avaliar qual o melhor tratamento para ele (afinal, ele é leigo e não tem o conhecimento técnico necessário para isso). O profissional deve se esforçar ao máximo para explicar ao paciente a importância da conduta, afinal o princípio da beneficência (e não o da autonomia) deve ser respeitado em primeiro lugar para alguns, e procurar sempre o consentimento, deixando clara a possibilidade de o paciente mudar de ideia. O paciente aqui descrito, por exemplo, retornou ao médico que o assistia convencido da conduta proposta, e foram passados alimentação por SNE e fonoterapia. Ele recuperou o estado nutricional e a segurança da alimentação oral.

Uma das limitações deste trabalho é nossa amostra, com poder de confiança de 90%. Por se tratar de estudo observacional, permite estabelecer associações, mas não definir a relação de causalidade e risco. Salientamos sua importância clínica da necessidade de iniciarmos discussão mais ampla entre todas as especialidades médicas sobre a decisão do paciente que não aceita nossa conduta, discutir os princípios bioéticos e aprendermos a conduzir os casos que nos desafiam e documentar a recusa do paciente. Não devemos desistir de fomentar o paciente com informações sobre seu real estado de saúde e suas expectativas diante da não aceitação do tratamento proposto, e as consequências da recusa, pois, muitas vezes, a autonomia do paciente é falseada por desinformação, e dúvidas sobre a proposta terapêutica, e uma vez esclarecido ele poderá mudar o seu posicionamento. Temos que nos esforçarmos para convencer os pacientes do melhor, e, caso contrário, darmos suporte para conduzi-los diante da recusa.

## CONCLUSÃO

O médico diante do paciente disfágico com risco de aspiração laringotraqueal e mentalmente capaz que não aceita a restrição da via oral enfrenta um conflito de valores éticos e morais. Nosso estudo demonstrou que a maioria dos participantes foram solidários à decisão do paciente e argumentaram que a autonomia é soberana isoladamente e/ou apesar dos riscos, ponderando os princípios da beneficência e não maleficência. Além disso, a decisão deve ser respeitada em indivíduos mentalmente capazes, assim como o direito de mudar de opinião. Entre os participantes não solidários, o balanço entre os princípios beneficência e não maleficência nortearam sua decisão, justificando o risco de piora clínica com aumento da morbimortalidade. Independente da ciência sobre a hierarquia dos princípios que regem o processo de decisão na nossa amostra, o principal princípio considerado neste processo foi o da autonomia entre os participantes solidários à decisão contrária à proposta oferecida. O princípio da justiça preponderou entre os participantes não solidários com mais tempo de formado. Preocupações éticas surgem diante da não aceitação da conduta proposta, surgindo a necessidade de formalizar a recusa no prontuário médico, por meio de documento de recusa informada, com finalidade protetiva legal.
